# The Campaign of Education

**Published:** 1896-09

**Authors:** 


### THE CAMPAIGN OF EDUCATION.

The campaign of education is on, and we see on every side its wholesome influences. The lessons it brings to us as a people have been slowly learned, and the cost has been dearly paid. We have looked in amazement upon the citizen who dared to eat half-cooked sausages or meat puddings, and thus contracted trichinosis from his foolhardiness. But with the utmost serenity we have watched thousands annually die from conditions arising from consuming, as a totally raw product, milk from diseased animals; from herds of cattle reeking in filth themselves; produced under such unsanitary conditions of the stables and cow-sheds that one could not enter them of a morning and remain even a few minutes without retreating again for fresh air; and this too after the stable had been opened up for the day. The attendants have deemed it unnecessary to even wash their hands, not to think of the possible need of cleansing the mammary glands, save by dipping the fingers in the milk and applying it to the teats, thus insuring the entrance to the milk of any dirt on these organs that might not be dry enough to fall off. The absence of light, not sunlight, has been deemed a wise method of contributing to the warmth of the stable; and then the flies were not so numerous, and the filthy accumulations were not so noticeable. The spring-houses were generally adjacent to the lowest part of the cow-yard, so that the drainage of the accumulated manure of the winter might saturate the earth adjacent to the little contributing rivulets which make up the spring's supply of water, and thus insure every possibility of contamination of this product which is so susceptible to every odor and so perfect a vehicle of transmission of so many products dangerous to the human system. But the light of better methods has been thrown on this state of affairs, and the eyes of a sanitary police system are opening to the needs of better work in the preparation, care, and sale of these products. The good work so well begun in Massachusetts, and so energetically pushed by determined men, has spread to the whole of the New England States, entered the Empire State, hesitates in New Jersey, is making rapid and permanent progress in the Keystone State, where every day adds to its advancement under the well-directed work of the Live-stock Sanitary Board; has crossed the Mississippi Valley and awakened the people of St. Louis, Kansas City, Sioux City, Minneapolis, and St. Paul, and many minor centres of closely aggregated people; fled to the Pacific coast, where much early missionary work had already been done, and now ramifies in a thousand directions from these chief centres of agitation. Speed the good work, everyone, for it is for the betterment of all. It will aid the producer by enhancing the value of his products; it will aid the public health boards in solving many of the perplexing outbreaks of dangerous infectious and contagious diseases; it will aid the physician in the management of his cases so often wholly dependent upon a nutrient diet, so largely found in milk when pure and wholesome; it will aid the veterinarian in more completely evolving the field of his greatest usefulness; and it will be a boon to our people in better health, in greater happiness, and contribute in every way to our higher and better advancement. A pure milk-supply is second only to a pure water supply, the most important problem for any people to solve.

---

At last horse-show authorities are awakening to another danger besides that of overlooking the necessity of rigid veterinary inspection of all breeding-classes. It is the use of shows by dealers who make large entries for the purpose of sale, and thus gain an advertisement for their horses and contribute very little to the real interest and objects of such exhibitions.

